# Supplementary material for: An Assessment of the Mechanophysical and Hormonal Impact on Human Endometrial Epithelium Mechanics and Receptivity
Source: Int J Mol Sci. 2024 Mar 27;25(7):3726. doi: 10.3390/ijms25073726 (PMC11011295; doi:10.3390/ijms25073726)
Supplement: Supplementary file 1 [file ijms-25-03726-s001.zip › ijms-2914247-supplementary.pdf]

Supplementary material

Table S1. List of antibodies used for immunofluorescence stainings.

	**Antigen**	**Species source, clonality**	**Order no, source of supply**	**Dilution**
Primary antibodies	Progesterone receptor (clone PgR636)	Mouse, monoclonal (IgG1)	M3569, DAKO GmbH, Jena, Germany	1:50
	Vimentin	Guinea pig polyclonal	GP 53, Progen, Germany	1:200
	ZO-1 (mid region)	Rabbit, polyclonal	UB280595, Thermo Fisher Scientific Inc., Waltham, MA, USA	1:200
Secondary antibodies	Alexa Fluor 488 goat anti-guinea pig IgG (H+L)	Goat	A-11073, Invitrogen, Thermo Fisher Scientific Inc., Waltham, MA, USA	1:500
	Alexa Fluor 555 goat anti-mouse IgG (H+L)	Goat	A-21424, Invitrogen	1:500
	Cy3 donkey anti-rabbit IgG (H+L)	Donkey $F(ab)_2$	711-166-152, Jackson/Dianova, West Grove, USA	1:500

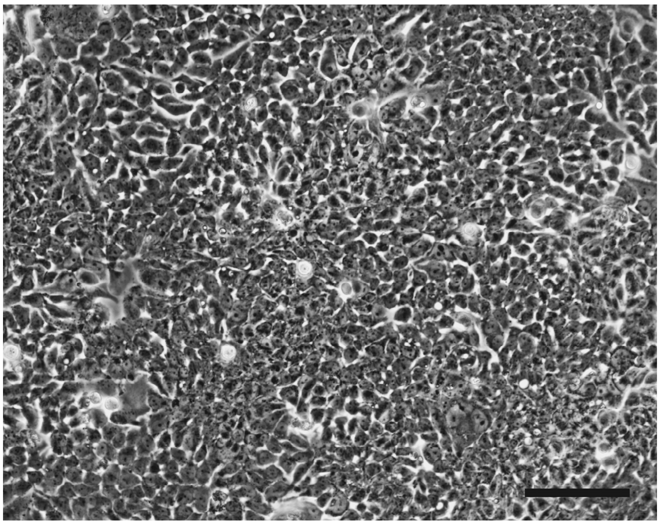

Figure S1. Ishikawa cells growing on Matrigel-coated plastic form confluent monolayers. The phase contrast image shows Ishikawa cells after 5 days in a polystyrene cell culture dish. The stiffness of polystyrene is ~3-4 GPa [1]. Scale bar = 100 μm.

[1] Miyake, K., Satomi, N. & Sasaki, S. Elastic modulus of polystyrene film from near surface to bulk measured by nanoindentation using atomic force microscopy. *Appl. Phys. Lett.* **89**, (2006).

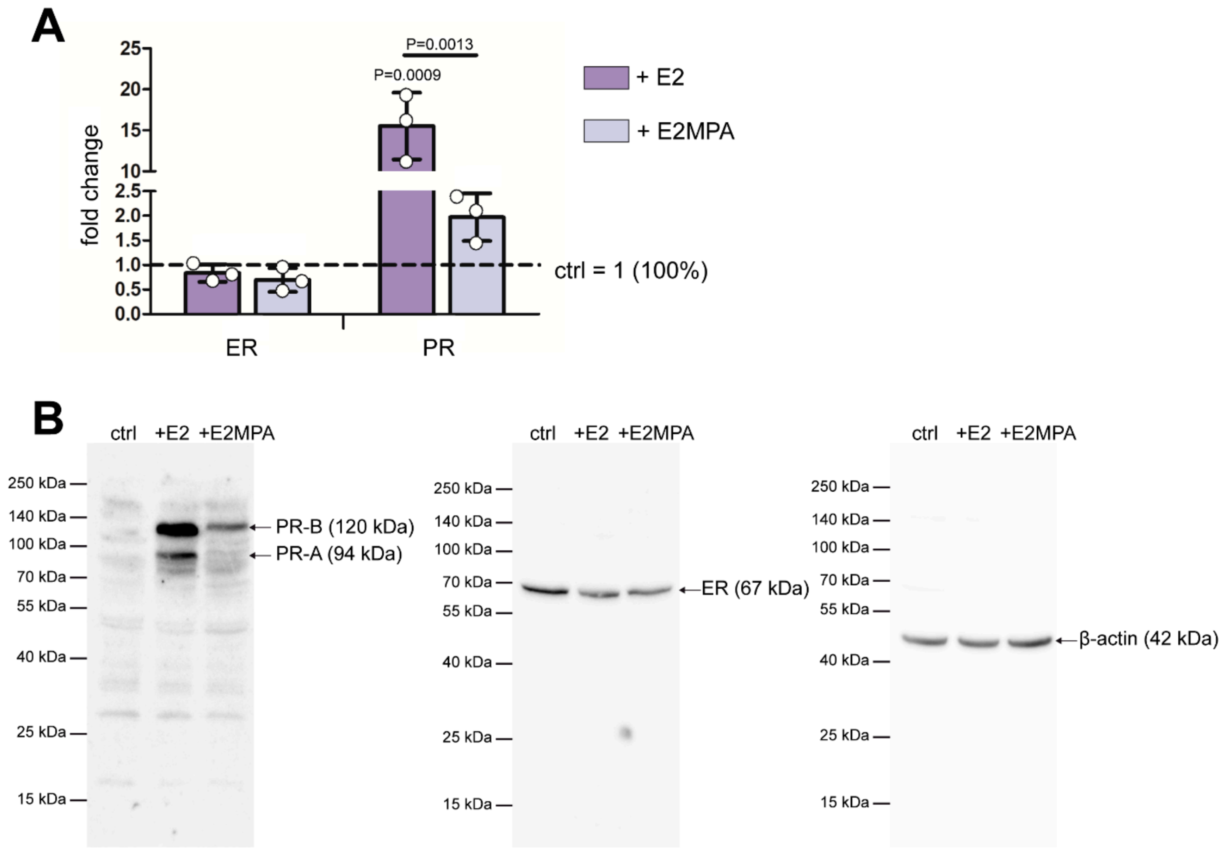

Figure S2. Progesterone receptor expression is regulated by steroid hormones. (**A**) The bar plot depicts immunoblot quantifications of the progesterone receptor (PR) and estrogen receptor (ER) in Ishikawa cells. Cells were either treated with estradiol (E2) or with a combination of estradiol and medroxyprogesterone acetate (E2MPA) for 72 hours. Protein levels were normalized against the non-treated control group. Note that the estrogen receptor protein levels are not regulated by hormone treatment whereas progesterone receptor protein levels are significantly upregulated in Ishikawa cells treated with estradiol. The data were obtained by 3 independent experiments (mean ± SD; one-way ANOVA with Bonferroni post-hoc test). (**B**) Representative immunoblots show the levels of PR and ER in Ishikawa cells. β-actin was used as a loading control.

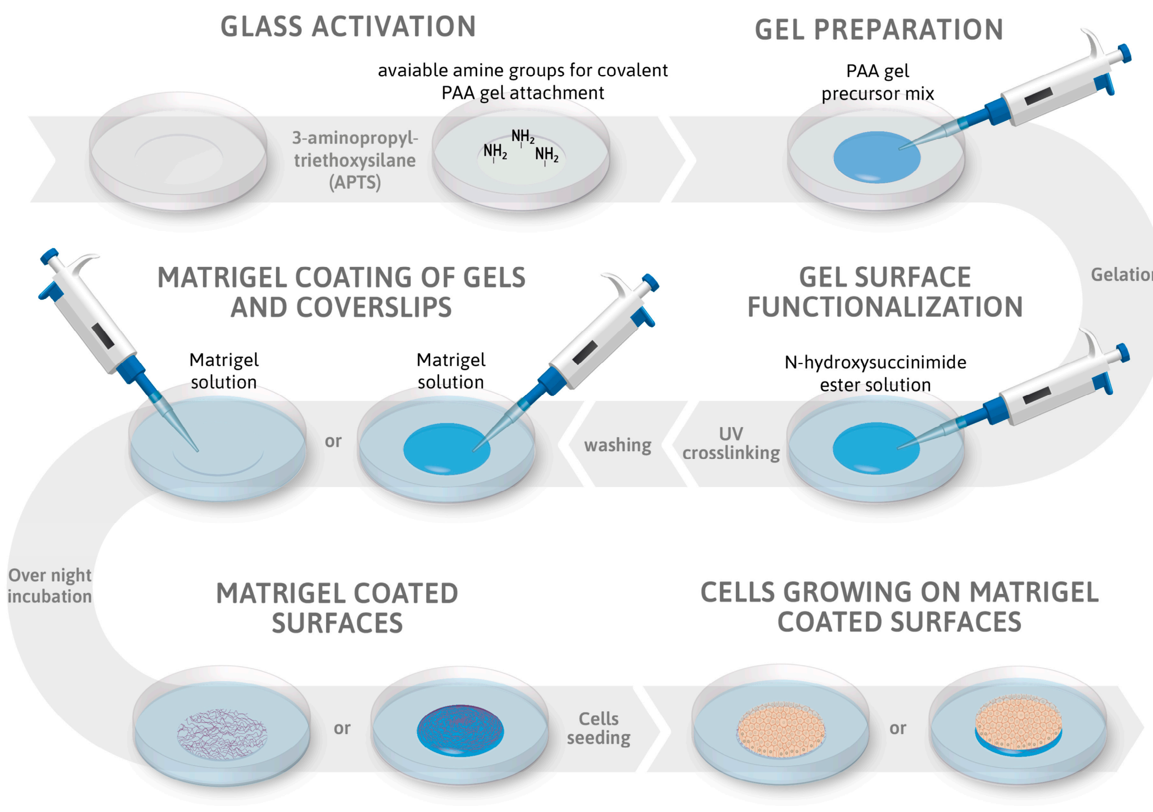

Figure S3. The scheme summarizes substrate preparation for cell seeding. The scheme depicts all main steps of the protocol (for further details see Materials and methods).

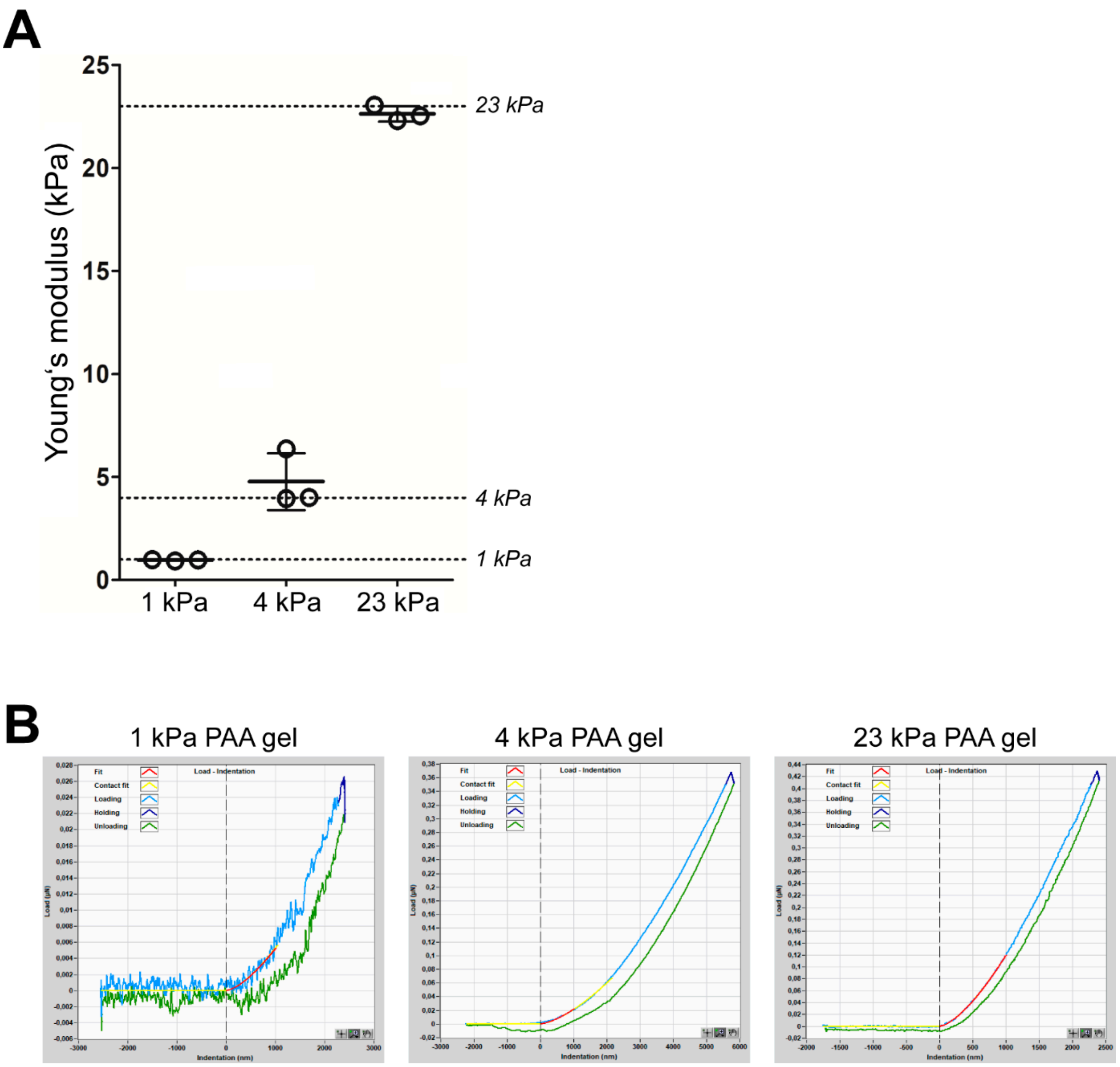

Figure S4. Stiffness of used polyacrylamide (PAA) gels. (**A**) Young's moduli of PAA gels obtained by nanoindentation measurement. The data were obtained by 3 independent experiments with 3 technical replicates per condition (mean ± SD). (**B**) Exemplary load-displacement curves of PAA gels of 1, 4 and 23 kPa stiffness.
